# 2D atomic crystal molecular superlattices by soft plasma intercalation

**DOI:** 10.1038/s41467-020-19766-x

**Published:** 2020-11-24

**Authors:** Lufang Zhang, Haiyan Nan, Xiumei Zhang, Qifeng Liang, Aijun Du, Zhenhua Ni, Xiaofeng Gu, Kostya (Ken) Ostrikov, Shaoqing Xiao

**Affiliations:** 1 Engineering Research Center of IoT Technology Applications (Ministry of Education), Department of Electronic Engineering, Jiangnan University, Wuxi, 214122 China; 2 School of Science, Jiangnan University, Wuxi, 214122 China; 3 Department of Physics, Shaoxing University, Shaoxing, 312000 China; 4 School of Chemistry and Physics and QUT Centre for Materials Science, Queensland University of Technology (QUT), Brisbane, QLD 4000 Australia; 5 Department of Physics and Key Laboratory of MEMS of the Ministry of Education, Southeast University, Nanjing, 211189 China; 6 CSIRO-QUT Joint Sustainable Processes and Devices Laboratory, P.O. Box 218, Lindfield, NSW 2070 Australia

**Keywords:** Synthesis and processing, Two-dimensional materials

## Abstract

Two-dimensional (2D) atomic crystal superlattices integrate diverse 2D layered materials enabling adjustable electronic and optical properties. However, tunability of the interlayer gap and interactions remain challenging. Here we report a solution based on soft oxygen plasma intercalation. 2D atomic crystal molecular superlattices (ACMSs) are produced by intercalating O_2_^+^ ions into the interlayer space using the plasma electric field. Stable molecular oxygen layer is formed by van der Waals interactions with adjacent transition metal dichalcogenide (TMD) monolayers. The resulting interlayer gap expansion can effectively isolate TMD monolayers and impart exotic properties to homo-(MoS_2_[O_2_]_*x*_) and hetero-(MoS_2_[O_2_]_*x*_/WS_2_[O_2_]_*x*_) stacked ACMSs beyond typical capacities of monolayer TMDs, such as 100 times stronger photoluminescence and 100 times higher photocurrent. Our potentially universal approach to tune interlayer stacking and interactions in 2D ACMSs may lead to exotic superlattice properties intrinsic to monolayer materials such as direct bandgap pursued for future optoelectronics.

## Abstract (web summary)

Two-dimensional (2D) atomic crystal superlattices offer technological opportunities beyond the reach of existing materials. Here, the authors produce 2D atomic crystal molecular superlattices by intercalating O_2_ molecules into the interlayer space of 2D materials using a soft plasma strategy.

## Introduction

Two-dimensional (2D) atomic crystal superlattices allow effective manipulation of stacking and coupling of atomic layers of diverse materials without restricting lattice matching due to the van der Waals forces between the adjacent layers. Therefore, such artificial 2D atomic crystal superlattices possess a wide range of adjustable electronic properties, offering technological opportunities, and applications beyond the reach of existing materials^1–5^. The most common fabrication methods based on layer-by-layer exfoliation and transfer are complex, laborious, and time-consuming, with limited yield and reproducibility^6–11^. Chemical vapor deposition (CVD) has been successfully applied to produce high-quality 2D heterostructures, bilayer, and multilayer transition metal dichalcogenides (TMDs), as well as lateral surface superlattices. However, CVD lacks growth and stacking precision and is thus impractical for high-order vertical superlattices^12–17^.

Recently, a new approach for the formation of vertical 2D superlattices has been demonstrated. Instead of growing or stacking layers of different 2D materials on top of each other, superlattice structures can be produced by the intercalation of selected 2D atomic crystals with alkali metal ions via electrochemical reactions^18–20^. Molecular intercalation is now regarded as a new and promising way to create the new class of stable superlattices in which monolayer atomic crystals alternate with ammonium bromide molecular layers^4^. However, the current state-of-the-art electrochemical approach is a wet process that often uses a whole 2D crystal as the source material and thus suffers from excessive material consumption. Furthermore, the alkali metal intercalation method usually requires the protection of inert gas to avoid the degradation of the properties of these superlattices, while the ammonium bromide molecule used for electrochemical intercalation is a toxic material.

To address the above issues, here we propose a soft oxygen plasma intercalation concept and demonstrate the 2D atomic crystal molecular superlattices where monolayer TMDs alternate with oxygen molecular layers. This dry method is effective for both mechanically exfoliated or CVD-grown TMD flakes (including MoS_2_, WS_2_, MoSe_2_, WSe_2_, and ReS_2_, etc.) with thicknesses ranging from 2 to 8 layers.

By using MoS_2_ as a model system, we demonstrate that plasma intercalation with oxygen molecular layers produces MoS_2_[O_2_]_*x*_ superlattices in which the interlayer distance increases from 0.6 to 0.9 nm compared to pure MoS_2_, thereby effectively decoupling the MoS_2_ monolayers. As such, the MoS_2_[O_2_]_*x*_ superlattices display extremely strong photoluminescence (PL) with an intensity approximately 100 times higher compared to pristine MoS_2_. We confirm the superlattice structure by means of PL spectroscopy, Raman spectroscopy, atomic force microscopy (AFM), X-ray photoelectron spectroscopy (XPS), transmission electron microscopy (TEM) as well as first-principle atomistic numerical simulations. The bilayer MoS_2_[O_2_]_*x*_/WS_2_[O_2_]_*x*_ superlattice lateral heterostructures show much better photoelectric performance than the pristine bilayer MoS_2_/WS_2_ lateral heterostructures.

## Results

### The schematic and chemical reaction of soft plasma intercalation

**Fig. 1:**
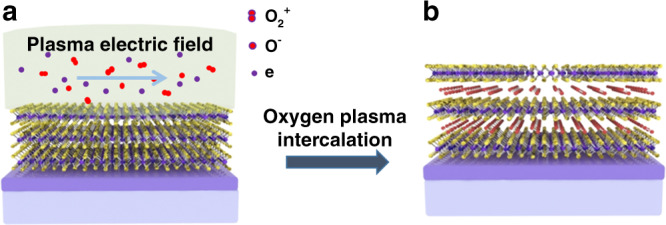
Soft oxygen plasma intercalation creates 2D ACMSs. **a** Schematic of the soft oxygen plasma treatment of few-layer MoS_2_ flakes, where the plasma-induced radial electrostatic field is parallel to the interlayer space of MoS_2_ flakes, and the oxygen plasma contains O_2_^+^ ions, O^−^ ions, O_2_ molecules, and electrons. **b** MoS_2_[O_2_]_*x*_ superlattices arise through the plasma-enabled oxygen intercalation and formation of the oxygen molecular layer within the interlayer spaces of the few-layer MoS_2_ flakes.

Figure 1 shows the schematic of fabricating monolayer-MoS_2_/O_2_-molecule superlattices by the soft oxygen plasma intercalation. The soft oxygen plasma was excited in the capacitive discharge mode (E-mode) of a planar low-frequency (2 MHz) inductively coupled plasma system, which is sketched in Supplementary Fig. 1. The capacitive coupling originating from the radial potential drop across the two ends of the planar induction coil generates the radial electrostatic field parallel to the substrate surface. This electrostatic field drives positive oxygen ions along the substrate surface and facilitates oxygen intercalation into the interlayer space between every two adjacent TMD layers. Furthermore, the E-mode discharge usually operates at very low input power (5–30 W) so that the ion density is too low to induce damage to the treated flakes. The chemical reaction of the soft oxygen plasma intercalation process consists of two half-reactions:

1 $${\mathrm{3O}}_2 + {\mathrm{e}}\mathop { \to }\limits^{{\mathrm{ionization}}} {\mathrm{2O}}_2^ + + {\mathrm{2O}}^ - + {\mathrm{e}},$$

2 $${\mathrm{MoS}}_{2} + x{\mathrm{O}}_{2}^ + + x{\mathrm{e}}\mathop { \to }\limits^{{\mathrm{intercalation}}} {\mathrm{MoS}}_{2}\left[ {\mathrm{O}}_{2} \right]_x.$$

The oxygen molecules formed in the interlayer space after oxygen ion intercalation may be stabilized via the van der Waals interaction with the adjacent MoS_2_ monolayers thereby forming the MoS_2_[O_2_]_*x*_ superlattices.

### Microscopic and optical characterization of MoS_2_[O_2_]_*x*_ superlattices

**Fig. 2:**
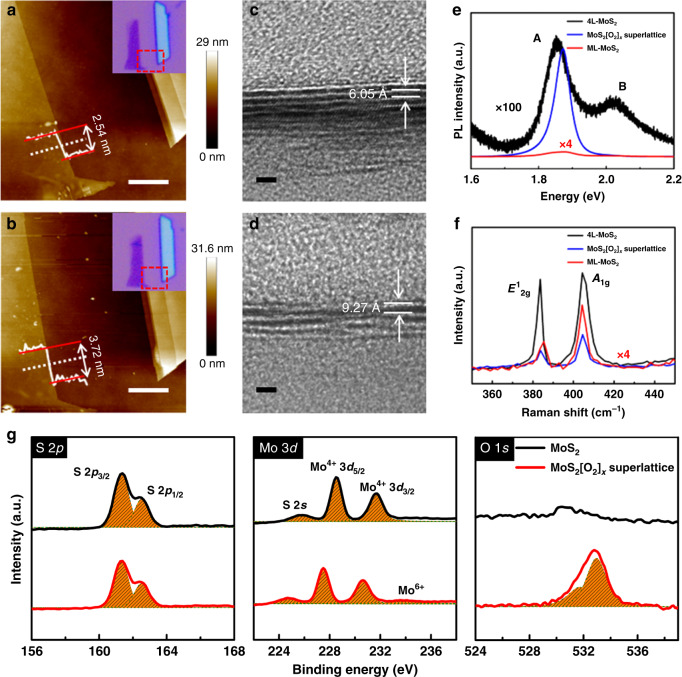
Structure and property evolution from MoS_2_ flake to MoS2[O2]_*x*_ superlattice. **a**, **b** AFM images of a pristine mechanically exfoliated four-layer MoS_2_ flake and the corresponding MoS_2_[O_2_]_*x*_ superlattice obtained by the 3 min long oxygen plasma intercalation. Insets are the corresponding optical images and height profiles along the dashed white lines. Scale bars: 1 μm. **c**, **d** Cross-sectional TEM images of a pristine MoS_2_ flake and a MoS_2_[O_2_]_*x*_ superlattice. Scale bars: 2 nm. **e**, **f** PL and Raman spectra of the MoS_2_ flake and MoS_2_[O_2_]_*x*_ superlattice in **a** and **b**, respectively, as well as the corresponding spectra (after multiplying by four) of monolayer MoS_2_. **g** High-resolution XPS spectra of S 2*p*, Mo 3*d,* and O 1*s* of the pristine MoS_2_ flake and MoS_2_[O_2_]_*x*_ superlattice.

Figure 2a, b displays the AFM images together with the corresponding optical images of a mechanically exfoliated MoS_2_ flake taken before and after 3 min of the oxygen plasma intercalation, respectively. AFM images reveal an apparent increase in the cross-sectional thickness from 2.54 to 3.72 nm. The slight change of the root-mean-square (RMS) roughness from 0.46 ± 0.14 nm to 0.74 ± 0.10 nm suggests that the surface of the sample was less affected by the 3 min oxygen plasma intercalation. The 1.18 nm increase in thickness, distributed across the 2.54 nm MoS_2_ flake consisting of 4 monolayers by assuming 0.65 nm as the thickness of a single S–Mo–S layer, corresponds to an average increase in each van der Waals gap of 3.93 Å. Further studies using TEM (Fig. 2c, d) show the distinct difference in microstructure between the pristine and treated flakes, giving a clearly resolved interlayer distance expansion ranging from 6.05 Å in the pristine sample (Fig. 2c) to 9.27 Å in the treated one (Fig. 2d). The deduced interlayer distance expansion (3.22 Å) is consistent with that (3.93 Å) obtained by the AFM measurements within the error margins, further validating the formation of MoS_2_[O_2_]_*x*_ superlattices.

More interestingly, as shown in Fig. 2e, the PL intensity of the MoS_2_ flake can be strongly enhanced by 100-fold after the oxygen plasma intercalation, along with a ~32 meV decrease of the full width at half-maximum (FWHM). For the pristine few-layer MoS_2_, the PL peak is well fitted to two peaks by using Lorentzian functions, which are assigned to A exciton (~1.83 eV) and B exciton (~1.96 eV), respectively^21,22^. Specifically, peak A is due to the direct electron–hole recombination in a neutral exciton at the K point, while peak B with higher energy is ascribed to the indirect electron–hole recombination in a neutral exciton at a lower valence band because of the spin–orbit coupling.

For the MoS_2_[O_2_]_*x*_ superlattice, however, the intensity of peak A is greatly enhanced by 100-fold while peak B vanishes. It is well-known that the layer number reduction from multilayer to monolayer can also lead to this variation in PL properties due to the indirect-to-direct bandgap transition^23^. The frequency differences between $E^1_{{\mathrm{2g}}}$ (in-plane vibrational mode) and *A*_1g_ (out-plane vibrational mode) peak of Raman spectra provide a reliable means to determine the number of layers^24,25^. The Raman spectra in Fig. 2f show a slight change of the frequency difference between $E^1_{{\mathrm{2g}}}$ and *A*_1g_ modes from 23.6 to 23.0 cm^−1^, indicating that the number of layers in the 4-layer (4 L) flake does not change. Both the decrease in Raman intensity of $E^1_{{\mathrm{2g}}}$ mode and the disorder between some of the layers in the TEM image (Fig. 2d) may be attributed to the disorder introduced in the MoS_2_ lattice by the incorporation of a small amount of oxygen-containing species as validated by the following XPS and first-principle calculation analysis. Another detailed experiment on the 6-layer MoS_2_ flake (Supplementary Fig. 2) reveals a thickness increase of 1.8 nm (corresponding to an average increase in each van der Waals gap of 3 Å), a PL enhancement of 60-fold (with peak A greatly enhanced and peak B disappearing) as well as similar Raman spectra behaviors.

We emphasize that both the astonishing enhancement of peak A and the vanishment of peak B are not the result of the indirect-to-direct bandgap transition from multilayer to monolayer due to a reduction in the layer numbers as proved by AFM, TEM, and Raman measurements. Instead, the observed indirect-to-direct bandgap transition owes to the formation of MoS_2_[O_2_]_*x*_ superlattices which can effectively isolate the MoS_2_ monolayers^26,27^.

In order to testify the universality of our soft oxygen plasma intercalation on MoS_2_ flakes, we performed similar experiments on 32 MoS_2_ flakes with different layer numbers ranging from 2 to 8 layers and list PL (including peak A position shift, FWHM, and intensity enhancement) and Raman (including .. position and *A*_1g_ position, FWHM) properties in Supplementary Tables 1 and 2, respectively. On average, the PL intensity (peak A) is increased by 53.1 times, and the FWHM of peak A decreases by 31 meV. Furthermore, the Raman statistical data shows that $E^1_{{\mathrm{2g}}}$ position hardly changes while *A*_1g_ position has a slight redshift of 0.7 cm^−1^ on average, revealing that the intralayer coupling is unaffected by the plasma intercalation, while the interlayer van der Waals coupling (out-of-plane vibration mode) becomes significantly weaker due to the isolation of every two adjacent MoS_2_ monolayers by the intercalated oxygen molecular layer. However, the van der Waals coupling between adjacent layers still exists and the corresponding Raman signals (*A*_1g_) can reflect the multilayer property as observed. On the other hand, the largely reduced van der Waals coupling plays a negligible effect on the PL effect as proved by the following calculated energy bands and therefore such MoS_2_[O_2_]_*x*_ superlattices exhibit an extremely strong PL behavior similar to that of MoS_2_ monolayer.

We also used energy-dependent XPS to probe the oxygen intercalation effect by comparing high-resolution XPS spectra of S 2*p*, Mo 3*d,* and O 1*s* between the pristine MoS_2_ flake and the MoS_2_[O_2_]_*x*_ superlattice, as shown in Fig. 2g. The strong peaks at 161.5 and 162.4 eV corresponding to S 2*p*_3/2_ and S 2*p*_1/2_ states, respectively, hardly change without any new peak emerging, indicating no S–O bonds formed during the oxygen plasma intercalation^28^. For O 1*s* and Mo 3*d*, however, the emergence of a strong peak at around 532 eV and a very small peak at 233.3 eV (apart from prominent Mo^4+^ 3*d*_3/2_ and Mo^4+^ 3*d*_5/2_ at 213.8 and 228.5 eV), respectively, reveal the fundamental change in microstructure after the oxygen plasma intercalation. The newly emerging O 1*s* peak can be decomposed into a peak at 531.7 eV (MoO_3_) and another peak at 532.9 eV (O_2_ molecule)^29^, while the small Mo 3*d* peak at 233.3 eV can be ascribed to Mo^6+^ (MoO_3_)^30^. In addition, doublet Mo 3*d* peaks also present a shift toward lower binding energy which indicates a shift in the Femi level toward the valence band and further validates the p-type doping by oxygen substitution at sulfur vacancies^31,32^. For the MoS_2_[O_2_]_*x*_ superlattice, the atomic percentage of O in the chemical form of MoO_3_ and O_2_ is estimated to be 0.348:0.652, meaning that most oxygen elements exist in O_2_ molecules and interact with MoS_2_ monolayer via van der Waals coupling to construct the superlattice in which MoS_2_ monolayers alternate with oxygen molecular layers.

### Mechanism of the soft oxygen plasma intercalation process

**Fig. 3:**
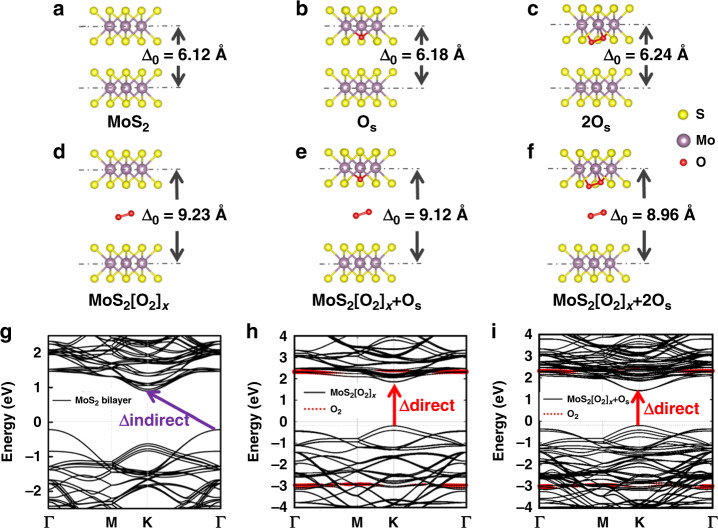
Calculated atomic configurations and energy band structures confirm interlayer space expansion and transition to direct optical bandgap. **a**–**e** Calculated atomic configurations of **a** the pristine MoS_2_ bilayer and four oxygen-involved MoS_2_ bilayer systems, namely MoS_2_ bilayer **b** with substitutional O site at S site (O_S_), **c** with substitutional 2O at 2S sites (2O_S_), **d** with oxygen molecule layer intercalated (MoS_2_[O_2_]_*x*_), **e** with both oxygen molecule layer intercalated and substitutional O site at S site (MoS_2_[O_2_]_*x*_ + O_S_), **f** with both oxygen molecule layer intercalated and substitutional 2O site at 2S sites (MoS_2_[O_2_]_*x*_ + 2O_S_). **g**–**i** Calculated band structures of the pristine MoS_2_ bilayer (**f**), the MoS_2_[O_2_]_*x*_ structure (**g**), and the MoS_2_[O_2_]_*x*_ + O_S_ structure, in which the atomic percentage of O in the chemical form of MoO_3_ and O_2_ is 1:2 (close to the XPS deduced ratio 0.348:0.652). The red dotted line in **g** corresponds to the energy state of the oxygen molecule.

In order to exclude potentially competing for oxygen bonding mechanisms that may expand the interlayer space, we carried out density functional theory (DFT) calculations on pristine MoS_2_ bilayer and five oxygen-incorporated MoS_2_ bilayer systems, namely MoS_2_ bilayer with substitutional O at S site (Os), with substitutional 2O at 2S sites (2Os), with oxygen molecule layer intercalated (MoS_2_[O_2_]_*x*_), with both oxygen molecule layer intercalated and substitutional O site at S site (MoS_2_[O_2_]_*x*_ + O_S_), with both oxygen molecule layer intercalated and substitutional 2O site at 2S sites (MoS_2_[O_2_]_*x*_ + 2O_S_), as shown in Fig. 3. For the MoS_2_[O_2_]_*x*_ + O_S_ structure, the atomic percentage of O in the chemical form of MoO_3_ and O_2_ is 1:2, which is very close to the XPS deduced value (0.348:0.652). For the MoS_2_[O_2_]_*x*_ + 2O_S_ structure, the atomic percentage of O in the chemical form of MoO_3_ and O_2_ is 1:1. Therefore, the MoS_2_[O_2_]_*x*_ + O_S_ model is the nearest to the real MoS_2_[O_2_]_*x*_ superlattices we obtained by such an optimum plasma intercalation process based on the above XPS results. The calculated band structures of three representative structures including MoS_2_ bilayer, MoS_2_[O_2_]_*x*_ and MoS_2_[O_2_]_*x*_ + O_S_ are displayed in Fig. 3g–i, respectively, while those of other structures including MoS_2_ monolayer, MoS_2_ bilayer with substitutional O at S site (Os), MoS_2_ bilayer with substitutional 2O at 2S sites (2Os), and MoS_2_ bilayer with both oxygen molecule layer intercalated and substitutional 2O site at 2S sites (MoS_2_[O_2_]_*x*_ + 2O_S_), are shown in Supplementary Fig. 3.

The pristine MoS_2_ bilayer exhibits the interlayer distance of 6.12 Å (Fig. 3a), while the interlayer distance increases slightly to 6.18 and 6.24 Å for the first two cases (Fig. 3b–c) with chemically bonded oxygen atoms (Mo–O bonds). Such an increase of the interlayer distance is not only inconsistent with the above experimental results but is also insufficient to cause an indirect-to-direct bandgap transition leading to the strong enhancement in PL intensity. Indeed, the calculated band structures of the first three cases (pristine MoS_2_ bilayer, MoS_2_ bilayer with O_S,_ and MoS_2_ bilayer with 2O_S_) exhibit an indirect bandgap as shown in Fig. 3g, Supplementary Fig. 3b, c. In contrast, for the MoS_2_[O_2_]_*x*_, MoS_2_[O_2_]_*x*_ + O_S_, and even MoS_2_[O_2_]_*x*_ + 2O_S_ modes, the interlayer distance can increase up to 9.23, 9.12, and 8.96 Å, respectively, due to the intercalation of oxygen molecular layer, fully complying with the above experimental results. Moreover, the calculated energy band structures of all the three modes display a direct bandgap (Fig. 3h, i and Supplementary Fig. 3d), which is comparable to that of MoS_2_ monolayer (Supplementary Fig. 3a), further supporting the conclusion that our soft oxygen plasma intercalation indeed can produce MoS_2_[O_2_]_*x*_ superlattice in which MoS_2_ monolayers alternate with oxygen molecular layers.

**Fig. 4:**
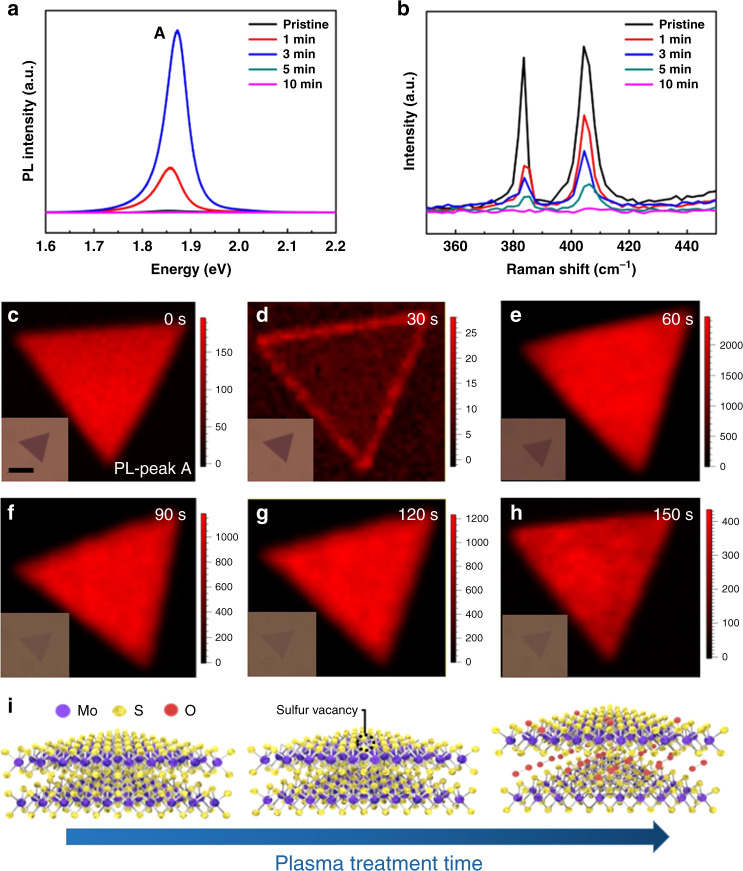
Treatment time-dependent PL and Raman properties and mechanism of the oxygen plasma intercalation. **a**, **b** Time-dependent PL (**a**) and Raman (**b**) spectra of the plasma-treated 4 L MoS_2_ flake. **c**–**h** Time-dependent PL intensity mapping of peak A of the plasma-treated CVD-grown MoS_2_ bilayer flake: **c** 0 s, **d** 30 s, **e** 60 s, **f** 90 s, **g** 120 s, and **h** 150 s. **i** Schematic physical picture of the oxygen plasma intercalation process.

Figure 4a, b presents the time-dependent PL and Raman properties of the 4 L MoS_2_ flake with the oxygen plasma treatment. One can deduce that the optimum treatment time for oxygen intercalation is 3 min. Once beyond this optimum treatment time, the crystal quality degrades significantly as reflected by both PL and Raman spectra. Similarly, the 6 L MoS_2_ flake displays the same evolution with an optimum treatment time of 4 min as reflected by the time-dependent PL and Raman properties in Supplementary Fig. 2. In order to have a more detailed understanding of the oxygen intercalation behavior, we chose one of the most common TMD materials, CVD-grown MoS_2_ bilayer, and recorded the time-dependent PL intensity mapping of peak A in Fig. 4c–h. The PL intensity first decreases at the first 30 s and then increases drastically and reaches a maximum (20 times as high as the pristine intensity) at 60 s. However, with the further increase in treatment time, the PL intensity decreases gradually.

By combining both the above experimental results and the DFT simulation, we obtain a clear physical picture of the oxygen plasma intercalation process as schematically illustrated in Fig. 4i. At the very beginning of the plasma treatment, the plasma irradiation can produce a small number of sulfur vacancies as well as the resultant formation of a small amount of Mo–O bonds via oxygen substitution at sulfur vacancies, thus suppressing the PL^33^. Thereafter, the O_2_^+^ ions generated by the plasma ionization (Eq. (1)) enter into the interlayer space driven by the force of the electrostatic field parallel to the interlayer space and subsequently form stable O_2_ molecules (Eq. (2)) there via the van der Waals interactions with the adjacent MoS_2_ monolayers. Consequently, the interlayer distance is expanding, effectively isolating the MoS_2_ and leading to the formation of MoS_2_[O_2_]_*x*_ superlattice with intriguing PL comparable with that of the corresponding intrinsic monolayer. Obviously, the interspace expansion effect prevails over the influence of plasma-induced Mo–O bonds at the optimum treatment time. However, once the treatment time is beyond this critical value, the influence of the plasma-induced Mo–O bonds may become more and more prominent, finally giving rise to the degradation of the crystal quality as reflected by both PL and Raman spectra. The time-dependent PL intensity mapping studies on vertically oriented MoS_2_ bilayer (Supplementary Fig. 4) shows no clue of any intercalation effect but the PL degradation, further proving the determining role of the parallel electrostatic field as well as the influence of plasma-induced Mo–O bonds. As shown in Supplementary Fig. 5, the MoS_2_[O_2_]_*x*_ superlattices based on bilayer, three-layer, four-layer, and six-layer MoS_2_ flakes exhibit good stability by maintaining its intriguing PL properties over 30 days.

### Photoelectric properties of MoS_2_[O_2_]_*x*_/WS_2_[O_2_]_*x*_ superlattices

**Fig. 5:**
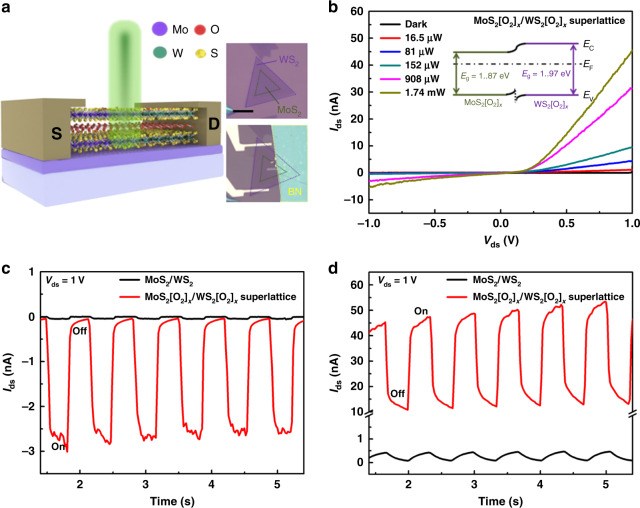
Evolution of photoelectric properties from MoS2/WS2 bilayer heterostructure to MoS_2_[O_2_]_*x*_/WS_2_[O_2_]_*x*_ superlattice heterostructure. **a** Schematic illustration and typical optical images of the MoS_2_[O_2_]_*x*_/WS_2_[O_2_]_*x*_ superlattice heterostructure. **b** *I*_ds_ − *V*_ds_ curves of the MoS_2_[O_2_]_*x*_/WS_2_[O_2_]_*x*_ superlattice heterostructure photodetector under 532 nm laser illumination with different power intensities. Inset is the energy band diagram of the MoS_2_[O_2_]_*x*_/WS_2_[O_2_]_*x*_ superlattice heterostructure. **c**, **d** The time-resolved photoresponse of both MoS_2_/WS_2_ bilayer heterostructure (before the plasma intercalation) and corresponding MoS_2_[O_2_]_*x*_/WS_2_[O_2_]_*x*_ superlattice heterostructure (after the optimum plasma intercalation) under **c** *V*_ds_ = −1 V (reverse bias) and **d** *V*_ds_ = 1 V (forward bias) with 532 nm (1.74 mW) illumination.

We have also studied the photoelectrical properties of the same MoS_2_/WS_2_ bilayer heterostructure and MoS_2_[O_2_]_*x*_/WS_2_[O_2_]_*x*_ superlattice heterostructure before and after the plasma intercalation process. Figure 5a, b displays the schematic illustration and the output characteristic (*I*_ds_ − *V*_ds_ curves) of the MoS_2_[O_2_]_*x*_/WS_2_[O_2_]_*x*_ superlattice heterostructure under the 532 nm laser illumination with different power intensities, respectively. The photocurrent (*I*_ds_) increases with the increase of the incident power, indicating that the number of photo-induced carriers increases as a result of the increase in the number of absorbed photons. The energy band diagram of such superlattice heterostructure shown in the inset of Fig. 5b exhibits an extremely small build-in electric field directing from MoS_2_[O_2_]_*x*_ to WS_2_[O_2_]_*x*_ due to their similar bandgap (1.87 eV for MoS_2_[O_2_]_*x*_ and 1.97 eV for WS_2_[O_2_]_x_) and identical n-type characteristics. This extremely small build-in electric field can be clearly reflected by the fact that the photocurrent (*I*_ds_) exhibits a faster increase with the increase in laser intensity under *V*_ds_ > 0 V (forward bias) compared to the case under *V*_ds_ < 0 V (reverse bias). Therefore, we choose two representative cases, the reverse bias (*V*_ds_ = −1 V) and the forward bias (*V*_ds_ = 1 V) to compare the photoelectrical properties between the same MoS_2_/WS_2_ bilayer heterostructure (before the plasma intercalation) and MoS_2_[O_2_]_*x*_/WS_2_[O_2_]_*x*_ superlattice heterostructure (after the optimum plasma intercalation), as shown in Fig. 5c, d, respectively. For the former case (*V*_ds_ = −1 V), the on/off ratio increases from ~10 to ~70 and the photocurrent increases by 70 times from 0.04 to 2.83 nA after the plasma intercalation process. For the latter case (*V*_ds_ = 1 V), the photocurrent increases by more than 100 times from 0.46 to 48.7 nA after the plasma intercalation process although the on/off ratio is hard to estimate due to the longer decaying time. This strikingly enhanced photoresponse can be attributed to the indirect-to-direct energy band transition after the plasma intercalation process^34,35^. We also fabricated a vertical 3L-MoS_2_/multilayer-WSe_2_ p–n heterojunction by the mechanical exfoliation and transfer method. The optimum plasma intercalation process can translate the top 3 L MoS_2_ flake with an indirect bandgap into MoS_2_[O_2_]_*x*_ superlattice with a direct bandgap and thus make the resultant self-powered photocurrent at *V*_ds_ = 0 V largely increased by 20 times (Supplementary Fig. 6), suggesting that our soft plasma intercalation technique has good stability and reproducibility in controlling the optical and electronic properties of these superlattices. The photoelectric performance can be further improved by using thicker superlattice lateral heterostructures with higher absorption.

Our approach may be expanded to intercalate diverse 2D mechanically exfoliated or CVD-grown TMD flakes, including WS_2_, MoSe_2,_ and ReS_2,_ etc. with thicknesses ranging from 2 to 8 layers, as evidenced by Supplementary Figs. 7–9, respectively. Similarly to the MoS_2_[O_2_]_*x*_ superlattice, WS_2_[O_2_]_*x*_ and MoSe_2_[O_2_]_*x*_ superlattices exhibit the same variation trend in the structural and optical properties compared to their pristine counterparts. However, for ReS_2_[O_2_]_*x*_ superlattice, no bandgap transition can be observed apart from the interlayer expansion. This result can be ascribed to the fact that both ReS_2_ monolayer and multilayer are direct bandgap semiconductors^36–38^. By considering the determining role of the electric field parallel to the interlayer space of TMDs, we expect that proper alignment of the plasma generated electric fields may lead to the observation of similar oxygen intercalation effects in other types of low-temperature plasmas.

## Discussion

In summary, the presented soft oxygen plasma intercalation generates 2D ACMSs where TMD monolayers alternate with oxygen molecular layers. This dry method is suitable for mechanically exfoliated or CVD-grown TMD flakes (including MoS_2_, WS_2_, MoSe_2_, and ReS_2,_ etc.) with thicknesses ranging from 2 to 8 layers. The physical mechanism can be ascribed to the O_2_^+^ ions entering the interlayer space driven by the parallel electric field and then forming stable O_2_ molecules via van der Waals interactions with the adjacent TMD monolayers. The interlayer distance can be largely expanded and becomes sufficient to effectively isolate the TMD monolayers as well as to almost suppress the interlayer coupling. These effects make superlattices such as MoS_2_[O_2_]_*x*_, WS_2_[O_2_]_*x*_, MoSe_2_[O_2_]_*x*_, and ReS_2_[O_2_]_*x*_ display monolayer characteristics. The bilayer MoS_2_[O_2_]_*x*_/WS_2_[O_2_]_*x*_ superlattice lateral heterostructures show much better photoelectric performance (100 times increased photocurrent) than the pristine bilayer MoS_2_/WS_2_ lateral heterostructures because of the indirect-to-direct energy band transition. Our studies thus provide a potentially universal approach to create such 2D ACMSs from pristine 2D nanomaterials and provide a generic platform for fundamental physics research and potential technological applications. Moreover, the 2D ACMSs with intrinsic monolayer characteristics such as a direct optical bandgap set an important milestone for future optoelectronics.

## Methods

### Sample preparation

Few-layer MoS_2_, WS_2_, MoSe_2_, and ReS_2_ flakes were exfoliated mechanically from corresponding bulk single crystals and deposited onto 300 nm SiO_2_/Si substrates. Bilayer MoS_2_ flakes were synthesized on 300 nm SiO_2_/Si substrates by chemical vapor epitaxy. Before mechanical exfoliation or CVD, all the substrates were first ultrasonically cleaned in acetone and alcohol, and then rinsed in deionized water and finally dried by nitrogen stream.

### Oxygen plasma intercalation

As shown in Supplementary Fig. 1, a home-made planar low-frequency (2 MHz) inductively coupled plasma system was applied to produce the MoS_2_[O_2_]_*x*_, WS_2_[O_2_]_*x*_, MoSe_2_[O_2_]_*x*,_ and ReS_2_[O_2_]_*x*_ superlattice. The input RF power was kept at as low as 20 W so that the plasma was excited in the E-mode of ICP, producing a radial electrostatic field parallel to the substrate surface. The working pressure was kept at 38 Pa by introducing O_2_ as the precursor gas with a flow rate of 10 sccm. The sample stage was kept rotating during the plasma treatment process to ensure uniform intercalation into the interlayer space of TMD flakes. Unless other specified, the samples were placed horizontally on the sample stage.

### Characterizations

Optical images were obtained by Optical Microscopy (Leica 4000M or Leica 2700M). AFM was carried out by using a Bruker Dimension ICON system in the tapping mode. Raman and PL measurements were recorded using a Renishaw Invia micro-Raman spectrometer with a 532 nm excitation laser. The laser power was lower than 0.2 mW to avoid any laser-induced heating. Raman spectra were measured using an 1800 l/mm grating to disperse the signal while PL ones were measured using a 600 l/mm grating. XPS (Thermo Scientific Esca lab 250Xi) with an Al-Kα (1486.6 eV) source was used to determine the chemical configurations of the TMD flake before and after oxygen plasma intercalation. The cross-sectional high-resolution transmission electron microscopy images were measured by using a Themis z TEM system with an accelerating voltage of 200 kV. All the measurements were performed at room temperature under ambient conditions.

### Device fabrication and photoelectrical measurements

Bilayer MoS_2_/WS_2_ lateral heterojunction flakes were synthesized on 300 nm SiO_2_/Si substrates by adjusting both the temperature and the carrier gas flow direction. Then, the exfoliated BN flakes were transferred onto the surface of bilayer MoS_2_/WS_2_ heterojunction flakes serving as an insulating layer via a dry transfer method with polydimethylsiloxane (PDMS). Subsequently, we fabricated Cr/Au (5 nm/50 nm) contact electrodes by using e-beam lithography and electron beam evaporation. After that, the bilayer MoS_2_/WS_2_ heterojunction devices were subjected to soft oxygen plasma intercalation and transformed into MoS_2_[O_2_]_*x*_/WS_2_[O_2_]_*x*_ superlattice heterostructures. The electrical and photoresponse characteristics of the same MoS_2_/WS_2_ bilayer heterostructure and MoS_2_[O_2_]_*x*_/WS_2_[O_2_]_*x*_ superlattice heterostructure before and after the plasma intercalation process were measured using a Keithley 2643B analyzer under dark and illuminated conditions in an atmospheric environment. Renishaw Invia micro-Raman spectrometer with a 532 nm excitation laser was employed to attain the spectral photocurrent response. For all the photocurrent measurements, the lasers were focused on the sample with a 50× objective (NA = 0.5) and the spot size of the light is about 1 μm, much smaller than the device channel length. Optical attenuators were used to change the power of the illuminated laser and a chopper with a frequency of 1 Hz was used to record the time-dependent photoresponse.

### First-principles calculations

The first-principles calculations were performed by the Vienna ab initio simulation package^39^ and the projected augmented-wave potential^40,41^. The exchange-correlation functional introduced by Perdew, Burke, and Ernzerhof^42^ within the generalized gradient approximation was applied in the calculations. The *p* semi-core states of Mo were described as valence electrons. For bilayer MoS_2_ we constructed a slab geometry with the insertion of a vacuum layer of 15 Å. The k-space mesh used was 12 × 12 × 1 for the slab structure. The energy cutoff for the plane-wave basis was set as 520 eV and the forces are relaxed less than 0.01 eV/Å. The positions of atoms were allowed to relax while the lattice constants of the unit cells were fixed to the experimental values.

## Supplementary information

Supplementary Information

## Data Availability

The data that support the findings of this study are available from the corresponding author on reasonable request.
