# Supplementary material for: 2D atomic crystal molecular superlattices by soft plasma intercalation
Source: Nat Commun. 2020 Nov 24;11:5960. doi: 10.1038/s41467-020-19766-x (PMC7686334; doi:10.1038/s41467-020-19766-x)
Supplement: Supplementary file 1 — Supplementary Information [file 41467_2020_19766_MOESM1_ESM.pdf]

Supplementary Information

2D atomic crystal molecular superlattices by soft plasma intercalation

Lufang Zhang[1,#], Haiyan Nan[1,#], Xiumei Zhang[2], Qifeng Liang[3], Aijun Du[4], Zhenhua Ni[5], Xiaofeng Gu[1], Kostya (Ken) Ostrikov[4,6], Shaoqing Xiao[1,*]

[1] *Engineering Research Center of IoT Technology Applications (Ministry of Education), Department of Electronic Engineering, Jiangnan University, Wuxi 214122, China.*

[2]*School of Science, Jiangnan University, Wuxi 214122, China.*

[3]*Department of Physics, Shaoxing University, Shaoxing 312000, China.*

[4]*School of Chemistry and Physics, Queensland University of Technology, Brisbane QLD 4000, Australia.*

[5]*Department of Physics and Key Laboratory of MEMS of the Ministry of Education, Southeast University, Nanjing 211189, China.*

[6]*CSIRO-QUT Joint Sustainable Processes and Devices Laboratory, P.O. Box 218, Lindfield NSW 2070, Australia.*

[#]*These authors contributed equally to this work.*

*Correspondence and requests for materials should be addressed to Shaoqing Xiao (email: xiaosq@jiangnan.edu.cn).

Soft plasma setup

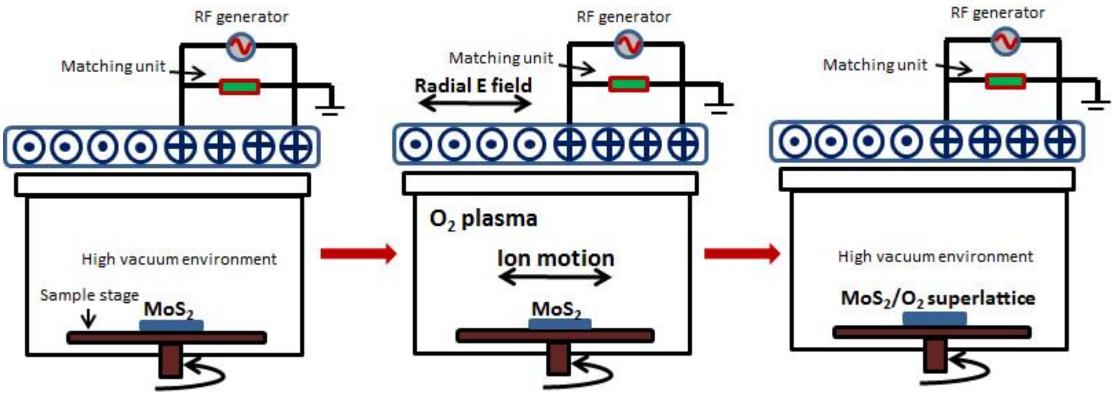

Supplementary Figure 1. The schematic of the soft plasma system together with the plasma treatment process to produce $MoS_2[O_2]_x$ superlattices.

Soft plasma mechanism: **Supplementary Figure 1** shows the schematic of the soft plasma system together with the plasma treatment process to produce $MoS_2[O_2]_x$ superlattices. As shown, a planar low-frequency (2 MHz) inductively-coupled plasma (ICP) source was applied to treat the MoS_2 flakes at room temperature. Although the plasma is generated by an inductive coil antenna, the plasma operates in the capacitive discharge mode (E-mode). The energy of RF generator was transferred from planar rectangular coil to plasma in both capacitive and inductive modes. The capacitive coupling originating from the radial potential drop across the two ends of the planar induction coil can produce radial electrostatic field parallel to the substrate surface while the mutual induction between the coil and plasma can induce the inductively-coupled electric field. At low input RF power of 5-30 W (in E mode), the induced electric field is much smaller than the radial electrostatic field due to low coil current and thicker plasma sheath. At this stage, direct ionization of feedstock gas by radial electrostatic field takes place but most of the generated electrons do not gain

sufficient kinetic energy to initiate further ionization collisions. This leads to low ionization rate and low electron density (of the order of magnitude of 10^9-10^{10} cm^{-3}) in this regime. As such, the ion density was too low to induce destructive ion bombardment onto the processed samples and this is how the soft plasma works.

From MoS_2 to $MoS_2[O_2]_x$ for 6-layer flake

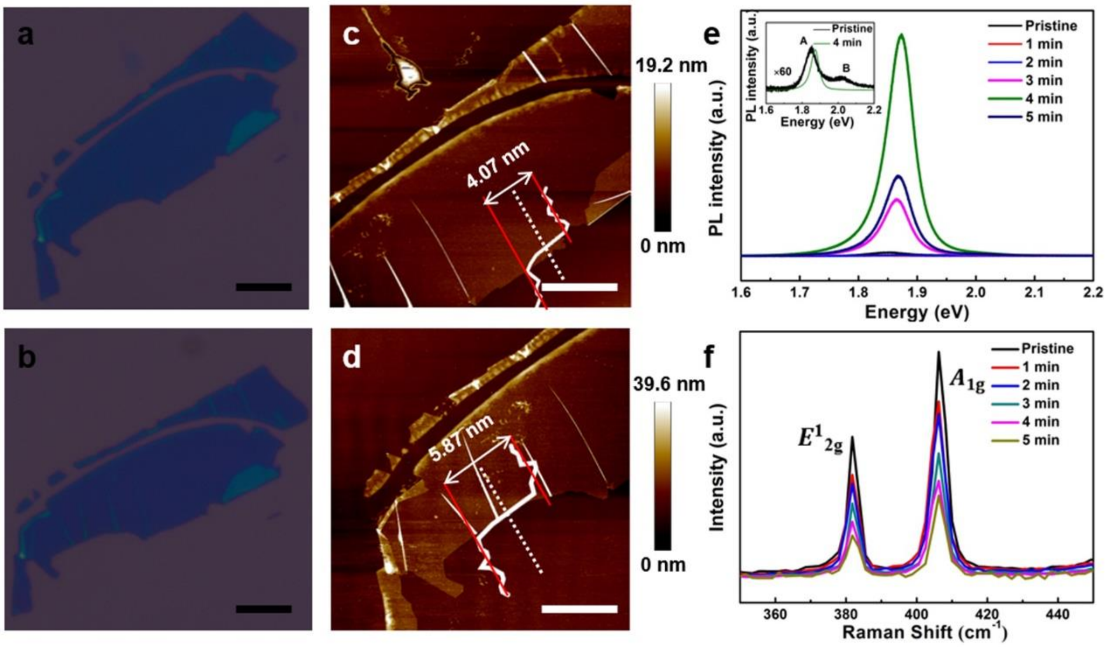

Supplementary Figure 2. (a), **(b)** Optical microscope images of a pristine mechanically exfoliated 6-layer MoS_2 flake and the corresponding $MoS_2[O_2]_x$ superlattice obtained by 4 min's oxygen plasma intercalation. Scale bars: 5 μm. **(c)**, **(d)** AFM images of the MoS_2 flake in **(a)** and the corresponding $MoS_2[O_2]_x$ superlattice in **(b)**, respectively. Scale bars: 5 μm. Time-dependent PL **(e)** and Raman **(f)** spectra of the plasma treated 6L MoS_2 flake. The inset in **(e)** shows PL spectra of the pristine MoS_2 flake and the corresponding $MoS_2[O_2]_x$ superlattice, highlighting the blueshift of the A exciton, the narrowing of the spectral linewidth, and the emergence of the symmetry in PL lineshape after plasma intercalation.

Statistical Photoluminescence and Raman Properties

Photoluminescence properties: **Supplementary Table 1** presents photoluminescence peak height, position, and the full width at half-maximum (FWHM) of the A exciton peak for 32 mechanically exfoliated flakes of MoS_2 with different layer numbers ranging from 2-8 layers before and after optimal oxygen plasma intercalation. Also, the blueshift of the position, the difference of FWHM (ΔFWHM) as well as the PL enhancement are listed. It is clear that all 32 flakes show an intriguingly enhanced PL intensity. According to the last line data, the PL is enhanced by 53.1 times, blueshifted by 16 meV, and the FWHM reduces by 31 meV on average. The evolutions of all these parameters indicate the indirect-to-direct bandgap transition. The large standard deviations in **Supplementary Table 1** can be attributed to the fact that the thickness and surface conditions may vary from sample to sample.

Supplementary Table 1. Statistical photoluminescence properties of 32 mechanically exfoliated flakes of MoS_2 with different layer numbers ranging from 2-8 layers before and after optimal oxygen plasma intercalation.

SN	Pre O_2 Intercalation			Post O_2 Intercalation					
	A Peak			A Peak					
	Height (Counts)	Center (eV)	FWHM (meV)	Height (Counts)	Center (eV)	FWHM (meV)	PL Blueshift (meV)	ΔFWHM (meV)	PL Enhancement
1	264	1.834	89	33645	1.856	59	-22	-30	127.4
2	312	1.849	97	25855	1.873	65	-24	-32	82.9
3	360	1.833	83	20815	1.862	57	-29	-26	57.8
4	380	1.860	99	10000	1.866	63	6	-36	26.3
5	378	1.839	85	14476	1.853	55	-14	-30	38.2

6	352	1.838	82	14417	1.863	55	-25	-27	41.0
7	429	1.856	90	12467	1.885	69	-29	-21	29.1
8	436	1.849	102	9864	1.873	68	-24	-34	22.6
9	547	1.857	95	45709	1.889	60	-33	-35	83.6
10	541	1.853	102	16561	1.871	55	-18	-47	30.6
11	455	1.856	91	12537	1.875	68	-19	-23	27.5
12	619	1.855	91	27489	1.870	55	-15	-36	44.4
13	400	1.849	95	9908	1.876	68	-27	-27	24.8
14	492	1.857	95	15724	1.880	59	-23	-36	32.0
15	532	1.849	97	17490	1.873	57	-24	-40	32.9
16	539	1.856	93	61985	1.867	59	-11	-34	115.0
17	532	1.853	99	16619	1.872	55	-9	-44	31.2
18	851	1.865	78	50443	1.865	103	1	25	59.3
19	896	1.850	100	18143	1.880	66	-30	-34	20.3
20	579	1.854	86	27668	1.850	55	4	-31	47.7
21	864	1.850	102	38950	1.868	73	-19	-29	45.1
22	416	1.838	91	19257	1.856	66	-19	-25	46.2
23	338	1.844	105	35522	1.859	57	-15	-48	105.2
24	608	1.858	91	19574	1.872	59	6	-32	32.2
25	475	1.857	92	15795	1.855	59	2	-33	33.2
26	619	1.862	88	18186	1.863	60	-1	-28	29.4
27	584	1.858	91	20635	1.874	61	-16	-30	35.3
28	510	1.848	92	17556	1.858	56	-10	-36	34.4
29	803	1.865	75	96199	1.884	42	-20	-33	119.8
30	879	1.849	97	18358	1.866	66	-17	-31	20.8
31	294	1.861	93	20888	1.871	55	-10	-38	71.1
32	807	1.849	97	122550	1.876	62	-27	-35	151.8
**Avg**	**534**	**1.852**	**92**	**28290**	**1.869**	**61**	**-16**	**-31**	**53.1**

Raman properties: **Supplementary Table 2** shows Raman scattering peak position and width of the $E^1{}_{2g}$ and A_{1g} Raman modes for corresponding 32 mechanically

exfoliated flakes of MoS_2 with different layer numbers ranging from 2-8 layers before and after optimal oxygen plasma intercalation. One can clearly observe that $E^1{}_{2g}$ position hardly changes while A_{1g} position has a slight red shift of 0.7 cm^{-1} on average, revealing that the interlayer van der Waals coupling (out-plane vibration mode) becomes weaker due to the isolation of every two adjacent MoS_2 monolayers by the intercalated oxygen molecule layers.

Supplementary Table 2. Statistical Raman properties of 32 mechanically exfoliated flakes of MoS_2 with different layer numbers ranging from 2-8 layers before and after optimal oxygen plasma intercalation. The samples correspond to those of **Supplementary Table 1** according to sample number (SN).

SN	$E^1{}_{2g}$ Peak				A_{1g} Peak			
	Pre O_2 Intercalation		Post O_2 Intercalation		Pre O_2 Intercalation		Post O_2 Intercalation	
	Centre	Fwhm	Centre	Fwhm	Centre	Fwhm	Centre	Fwhm
1	382.9	4.7	382.8	3.6	405.5	5.3	404.8	5.3
2	383.4	4.5	383.6	5.5	405.9	5.7	405.0	7.0
3	382.3	5.1	383.1	6.0	405.2	6.4	406.5	7.7
4	383.8	3.6	383.9	4.6	405.9	6.6	405.4	7.2
5	383.3	4.2	383.6	3.4	405.3	7.2	404.7	4.7
6	383.9	5.6	383.8	3.4	406.1	9.9	405.2	4.7
7	384.2	3.4	383.2	3.4	406.1	7.2	405.1	5.1
8	383.4	3.4	383.5	3.2	405.9	7.3	404.7	5.1
9	384.3	3.5	383.8	3.2	405.9	7.1	404.3	5.9
10	383.5	3.4	382.9	1.9	405.6	7.8	404.5	4.7
11	384.0	2.7	383.2	3.8	405.7	5.5	405.4	7.2
12	383.6	3.4	383.1	2.3	405.8	6.8	404.5	4.9
13	383.9	3.3	382.8	4.4	405.6	5.5	405.7	8.1
14	384.1	3.8	384.1	5.1	406.1	6.2	405.6	5.1

15	384.2	3.4	384.6	2.9	406.1	6.6	405.9	4.1
16	384.0	3.1	384.0	3.4	406.8	5.7	406.2	5.0
17	384.1	3.0	384.5	3.2	406.6	5.6	405.7	5.1
18	384.6	3.3	384.4	3.7	406.2	7.3	405.6	4.5
19	383.9	3.7	384.3	2.7	405.7	6.9	405.1	4.6
20	384.1	3.3	384.0	2.1	405.0	5.6	404.6	4.6
21	384.3	4.1	383.3	3.5	406.9	6.6	405.8	5.7
22	384.1	3.6	383.5	3.9	405.9	5.9	404.3	4.6
23	384.1	4.1	383.4	5.2	405.7	6.5	404.8	5.5
24	384.6	3.1	384.4	2.4	406.8	5.6	405.9	5.2
25	384.1	3.1	384.0	2.4	407.7	5.5	407.0	4.8
26	384.7	3.6	383.8	3.9	406.6	7.4	405.8	4.9
27	384.3	3.4	383.1	2.6	406.1	6.5	405.2	5.2
28	383.9	3.4	383.9	2.7	405.3	6.3	404.5	4.7
29	384.1	3.0	384.8	2.8	407.0	5.3	406.9	4.1
30	384.0	3.2	382.8	5.5	405.7	5.5	404.6	7.9
31	383.2	3.1	384.0	3.2	405.1	7.2	404.9	4.7
32	384.1	3.2	384.0	3.5	406.7	5.5	406.0	7.9
**Avg**	**383.9**	**3.6**	**383.7**	**3.5**	**406.0**	**6.4**	**405.3**	**5.5**

Density functional theory calculations on other MoS_2 models

It is clear from **Supplementary Figure 3** that MoS_2 monolayer exhibits a direct bandgap, while both the two models with chemically bonded oxygen atoms (O_S and $2O_S$) display an indirect bandgap. Once the oxygen species are intercalated into the interlayer space and form stable O_2 molecules there via the van der Waals interactions with the adjacent MoS_2 monolayers, the resulting systems including $MoS_2[O_2]_x$, $MoS_2[O_2]_x+O_S$ and even $MoS_2[O_2]_x+2O_S$ modes exhibit a direct bandgap.

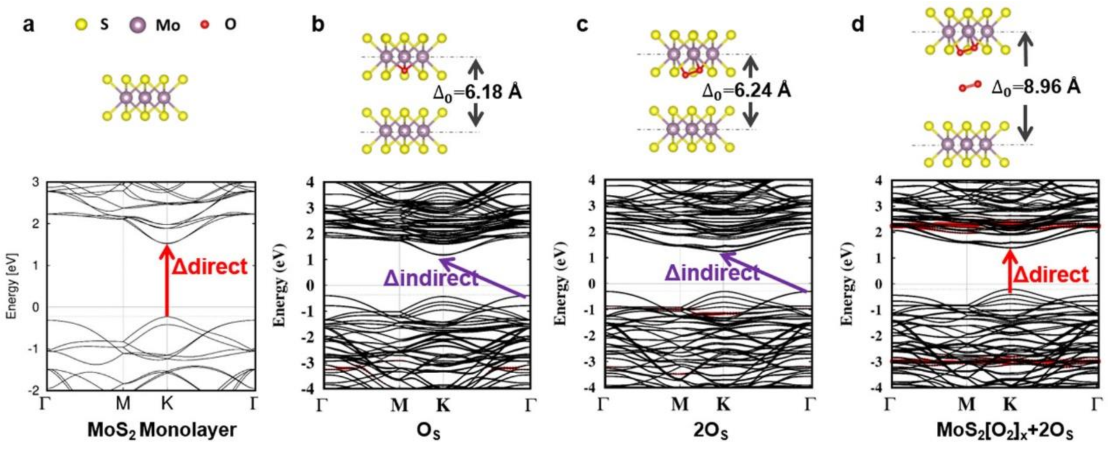

Supplementary Figure 3. Calculated atomic configurations and energy band structures for (a) MoS_2 monolayer, (b) MoS_2 bilayer with substitutional O site at S site (O_S), (c) MoS_2 bilayer with substitutional 2O at 2S sites ($2O_S$) and (d) MoS_2 bilayer with both oxygen molecule layer intercalated and substitutional 2O site at 2S sites ($MoS_2[O_2]_x+2O_S$).

PL mapping studies on vertically orientated MoS_2 bilayer

For comparison, we placed another CVD-grown MoS_2 bilayer flake vertically onto the substrate surface and recorded the time-dependent PL intensity mapping of peak A in **Supplementary Figure 4a-4e**. The PL intensity degrades significantly as long as the treatment time exceeds 30 s. This can be well understood since the plasma irradiation can produce sulfur vacancies and the resultant formation of Mo-O bonds and thus suppress the PL. However, the PL properties show no sign of recovery or getting better, indicating no clue of any intercalation effect. Unlike the case of horizontal placement of the MoS_2 bilayer sample where the plasma-induced electrostatic field is parallel to the interlayer space, the plasma-induced electrostatic field is perpendicular to the interlayer space in this controlled case. Therefore, we can conclude that the parallel electrostatic field is critical in achieving plasma

intercalation and the resultant 2D ACMSs.

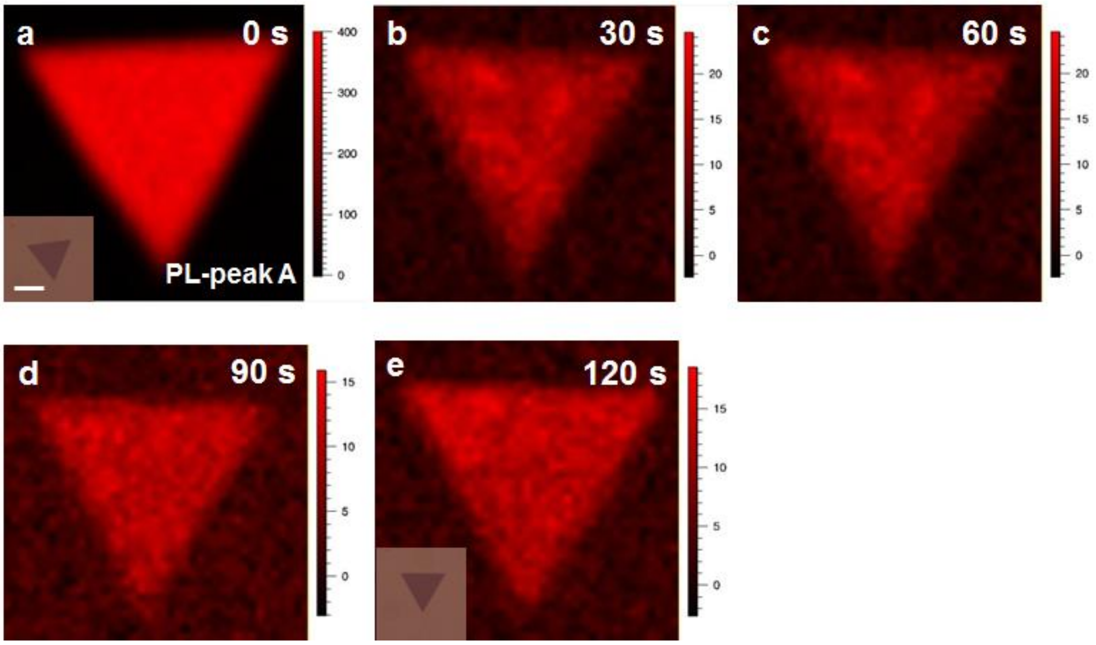

Supplementary Figure 4. Time-dependent PL intensity mapping of peak A of MoS_2 bilayer placed vertically onto the substrate surface: (**a**) 0 s; (**b**) 30 s; (**c**) 60 s; (**d**) 90 s; (**e**) 120 s. Insets are the corresponding optical microscope images. Scale bars: 20 μm.

Time durability of the $MoS_2[O_2]_x$ superlattice

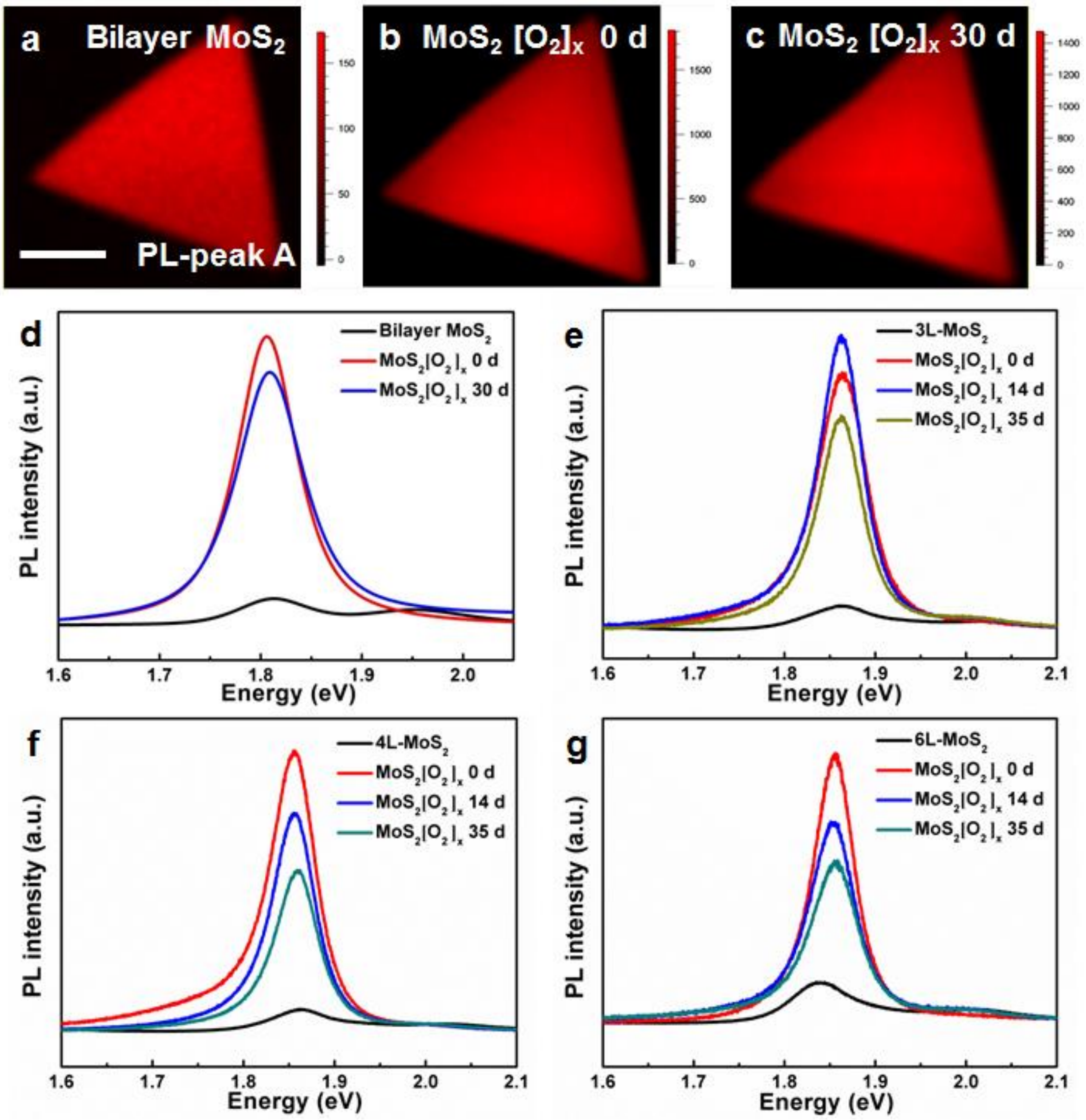

Supplementary Figure 5. PL intensity mapping of the pristine MoS_2 bilayer (**a**) and the corresponding $MoS_2[O_2]_x$ superlattice exposing to air for 0 days (**b**) and 30 days (**c**). Scale bars: 20 μm. (**d**) Single point PL spectra of the pristine MoS_2 bilayer (**d**), 3L-MoS_2 (**e**), 4L-MoS_2 (**f**), 6L-MoS_2 (**g**) and the corresponding $MoS_2[O_2]_x$ superlattices exposing to air for different durations.

Photoresponse of plasma treated vertical MoS_2/WSe_2 junction

We also fabricated a vertical 3L-MoS_2/multilayer-WSe_2 p-n heterojunction by mechanical exfoliation and transfer method as shown in **Supplementary Figure 6a**. The 3L-MoS_2 is on top of the multilayer WSe_2. The PL intensity mappings of peak A for both WSe_2 (1.55 eV) and MoS_2 (1.85 eV) are displayed in **Supplementary Figure 6b** and **6c**, respectively. The single PL and Raman spectra on MoS_2-only area, WSe_2-only area and the overlapping area of MoS_2 and WSe_2 are shown in **Supplementary Figure 6d** and **6e**, respectively. One can observe that both MoS_2-only and WSe_2-only areas exhibit their intrinsic strong PL peaks at 1.85 and 1.55 eV, respectively. However, these two PL signals are significantly reduced in the overlapping heterojunction region, and this can be attributed to the charge transfer between n-type MoS_2 and p-type WSe_2[5]. The characteristic Raman peaks of MoS_2, $E^1{}_{2g}$ and A_{1g}, locate at 382.3 and 404. 8 cm^{-1}, respectively. The frequency difference is 22.5 cm^{-1}, revealing that MoS_2 is three layer in thickness. For multilayer WSe_2, in addition to the $E^1{}_{2g}$ and A_{1g} peaks at 248.0 cm^{-1} and 256.2 cm^{-1}, respectively, there are also $B^2{}_g$ peak at 307.7 cm^{-1}, A_{1g}+LA peak at 372.4 cm^{-1} and 2A_{1g}-LA peak at 393.8 cm^{-1}. The Raman intensities of both MoS_2 and WSe_2 related peaks in the overlapping heterojunction region become weaker due to the occurrence of charge transfer between these two materials.

Different from the lateral MoS_2/WS_2 high-low n-n heterojunction, the vertical 3L-MoS_2/multilayer-WSe_2 p-n heterojunction possesses a strong built-in electric field, which can separate the photo-generated carriers effectively and produce a remarkable photocurrent even at V_{ds}=0 V. **Supplementary Figure 6f** shows the time-dependent self-powered (under zero bias V_{ds}=0 V) photocurrent of the vertical 3L-MoS_2/multilayer-WSe_2 p-n heterojunction with the increase in plasma treatment time. The photocurrent first decreases slightly at the very beginning of plasma treatment (30 s) and then increases significantly and reaches a maximum at 120 s. The

photocurrent increases by more than 20 times from 0.72 nA to 14.1 nA. The 120 s plasma intercalation is optimal to translate the top 3L MoS_2 flake with an indirect bandgap into $MoS_2[O_2]_x$ superlattice with a direct bandgap and thus promote significantly the photocurrent. However, such plasma treatment may have little effect on the bottom thick WSe_2 flake. The self-powered photocurrent also exhibits a fast increase with the increase in laser intensity as shown in **Supplementary Figure 6g**, suggesting that our soft plasma intercalation technique has good stability and reproducibility in controlling the optical and electronic properties of these superlattices.

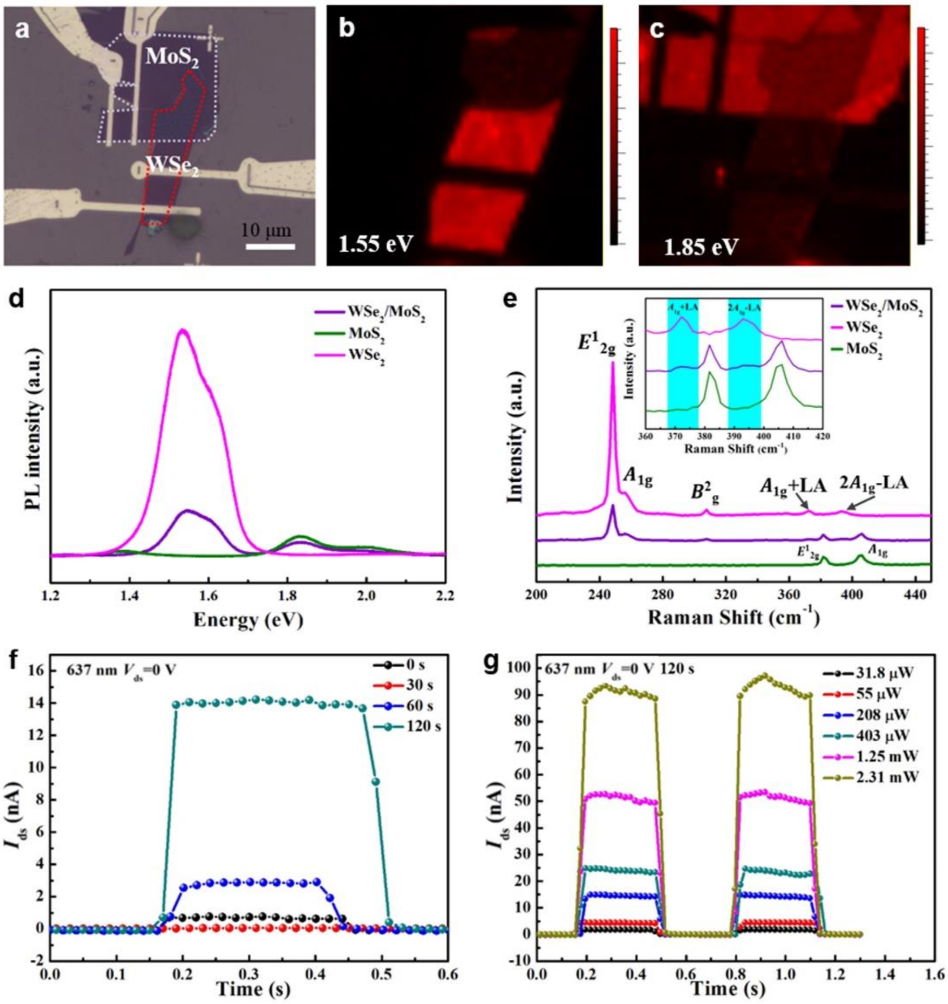

Supplementary Figure 6. (**a**) Optical image of the vertical 3L-MoS_2/multilayer-WSe_2 p-n heterojunction device. The PL intensity mappings of the corresponding MoS_2/WSe_2 heterojunction device at (**b**) WSe_2 A peak (1.55 eV) and (**c**) MoS_2 A peak (1.85 eV). Single PL (**d**) and Raman (**e**) spectra on MoS_2-only, WSe_2-only and the overlapping areas. The inset shows an enlarged region between 360 and 420 cm^{-1}. (f) The time-dependent photoresponse of the vertical MoS_2/WSe_2

junction as a function of the plasma treatment time. (g) The time-dependent photoresponse of the vertical MoS_2/WSe_2 junction subjected to the optimum plasma intercalation (120 s) under 637 nm laser illumination with different power intensities.

Universality for other TMDs

Such soft plasma intercalation strategy can be extended to diverse 2D mechanically exfoliated or CVD-grown TMD flakes including WS_2, $MoSe_2$ and ReS_2 etc. with thicknesses ranging from 2 to 10 layers, as evidenced by **Supplementary Figure 7**, **8**, **9**, respectively. From **Supplementary Figure 7**, one can clearly observe an apparent increase in the cross-sectional thickness from 2.74 nm (pristine 4L WS_2 flake) to 3.92 nm ($WS_2[O_2]_x$ superlattice), corresponding to an average increase in each van der Waals gap of 3.93 Å. The PL spectra highlight an enhancement of 43 times in PL intensity, the blueshift of the A exciton, the narrowing of the spectral linewidth, and the emergence of the symmetry in PL lineshape after the formation of $WS_2[O_2]_x$ superlattice. The Raman spectra show that ${E^1}_{2g}$ position hardly changes while A_{1g} position has a slight red shift, revealing that the interlayer van der Waals coupling (out-plane vibration mode) becomes weaker due to the isolation of every two adjacent WS_2 monolayers by the intercalated oxygen molecule layers.

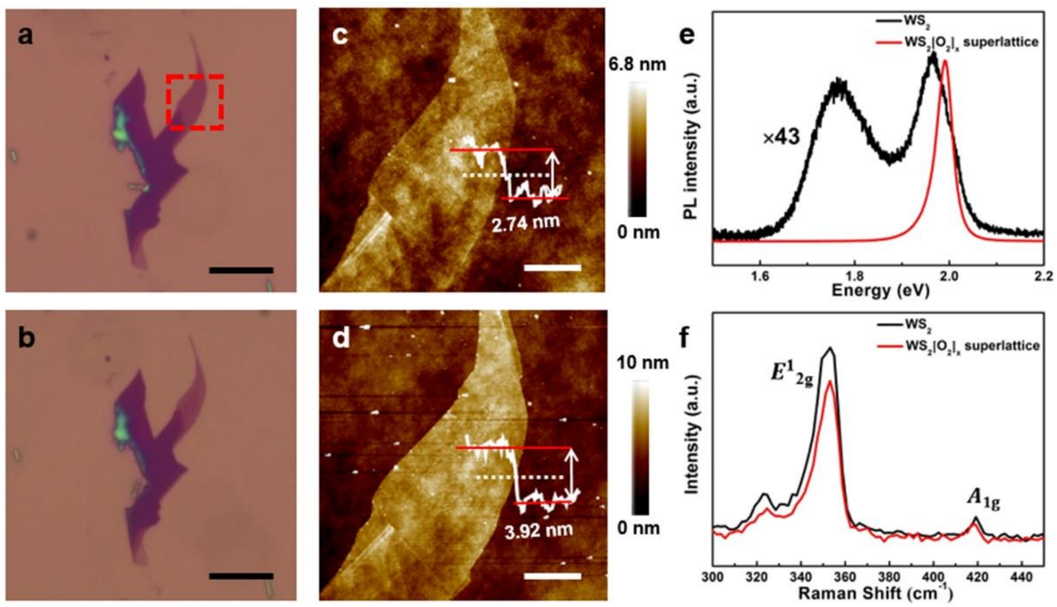

Supplementary Figure 7. (**a**), (**b**) Optical microscope images of a pristine mechanically exfoliated 4L WS_2 flake and the corresponding $WS_2[O_2]_x$ superlattice obtained by 3 min's oxygen plasma intercalation. Scale bars: 10 µm. (**c**), (**d**) AFM images of the WS_2 flake in **(a)** and the corresponding $WS_2[O_2]_x$ superlattice in **(b)**, respectively. Scale bars: 2 µm. PL (**e**) and Raman spectra (**f**) of the pristine WS_2 flake and the corresponding $WS_2[O_2]_x$ superlattice.

From **Supplementary Figure 8**, one can clearly observe an apparent increase in the cross-sectional thickness from 1.92 nm (pristine 3L $MoSe_2$ flake) to 2.83 nm ($MoSe_2[O_2]_x$ superlattice), corresponding to an average increase in each van der Waals gap of 4.55 Å. The PL spectra highlight an enhancement of 40 times in PL intensity, the blueshift of the A exciton, and the narrowing of the spectral linewidth after the formation of $MoSe_2[O_2]_x$ superlattice. The Raman spectra also show that $E^1{}_{2g}$ position hardly changes while A_{1g} position has a slight red shift, revealing that the interlayer van der Waals coupling (out-plane vibration mode) becomes weaker due to the isolation of every two adjacent $MoSe_2$ monolayers by the intercalated oxygen molecule layers.

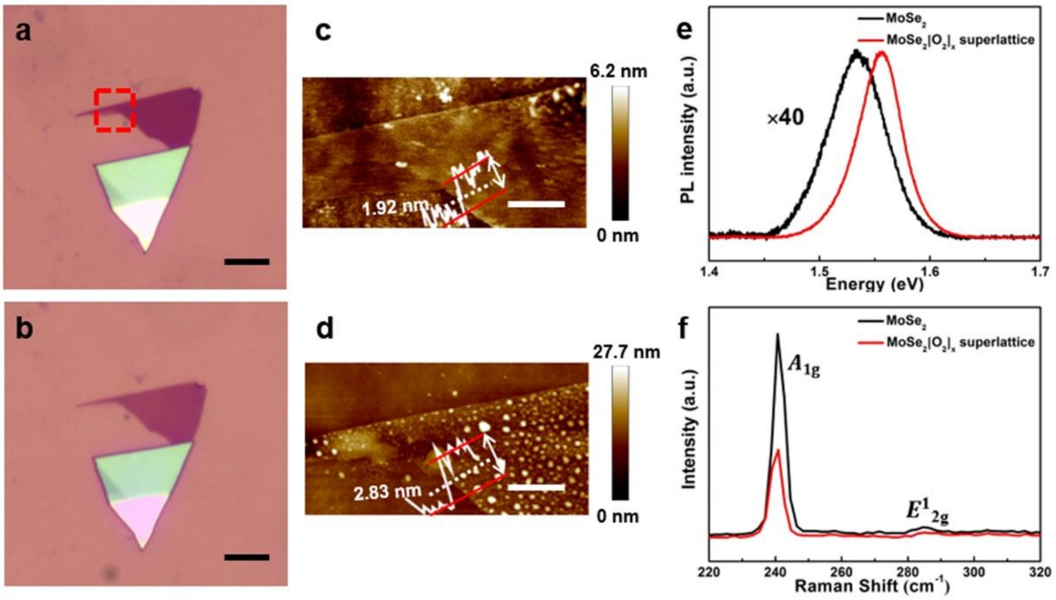

Supplementary Figure 8. (**a**), (**b**) Optical microscope images of a pristine mechanically exfoliated 3L $MoSe_2$ flake and the corresponding $MoSe_2[O_2]_x$ superlattice obtained by 2 min's oxygen plasma intercalation. Scale bars: 5 µm. (**c**), (**d**) AFM images of the $MoSe_2$ flake in **(a)** and the corresponding $MoSe_2[O_2]_x$ superlattice in **(b)**, respectively. Scale bars: 1 µm. PL (**e**) and Raman spectra (**f**) of the pristine $MoSe_2$ flake and the corresponding $MoSe_2[O_2]_x$ superlattice.

For ReS_2, we can also obtain an obvious increase in the cross-sectional thickness from 2.62 nm (pristine 4L ReS_2 flake) to 3.65 nm ($ReS_2[O_2]_x$ superlattice) as shown in **Supplementary Figure 9**, corresponding to an average increase in each van der Waals gap of 3.43 Å. In contrast to the above cases including $MoS_2[O_2]_x$, $WS_2[O_2]_x$ and $MoSe_2[O_2]_x$ superlattices, the PL spectra show no enhancement but a degradation in PL intensity after the formation of $ReS_2[O_2]_x$ superlattice. This could be well understood because both ReS_2 monolayer and multilayer are direct bandgap semiconductors[5-7] even though the resultant $ReS_2[O_2]_x$ superlattice contains ReS_2 monolayers isolated by the oxygen molecule layers in it. The Raman spectra display 18 vibration modes due to the intrinsic low symmetry and distorted 1T structure of

ReS_2[8,9] as shown in **Supplementary Figure 9f**. Among them, mode I and II located at 136.8 and 143.5 cm^{-1} are assigned to A_g-like modes corresponding to the out-of-plane vibrations of Re atoms, while modes III–VI located at 150.8, 160.7, 211.86 and 237.8 cm^{-1} are E_g-like modes corresponding to the in-of-plane vibrations of Re atoms. The remaining 12 higher-frequency Raman modes are mostly due to the vibrations of S atoms[10]. It was reported that[11] the frequency difference of mode III and I decreases as the layer number increases, providing a convenient method for identifying the layer number. No change in the positions of modes III and I can be observed, further indicating no change in the layer number after such soft plasma intercalation.

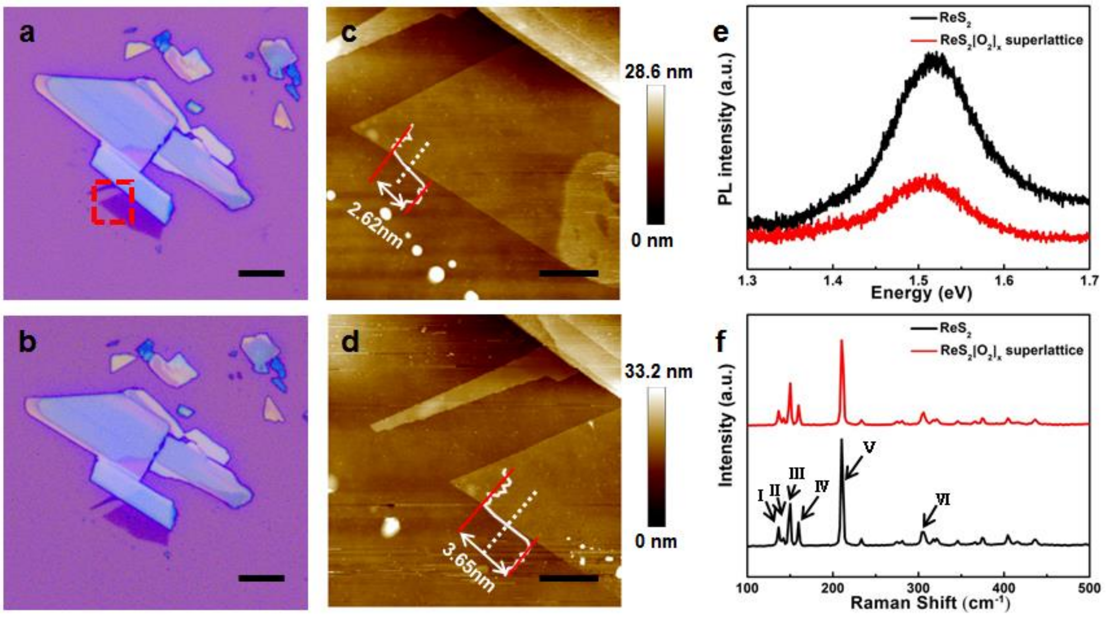

Supplementary Figure 9. (**a**), (**b**) Optical microscope images of a pristine mechanically exfoliated 4L ReS_2 flake and the corresponding $ReS_2[O_2]_x$ superlattice obtained by 3 min's oxygen plasma intercalation. Scale bars: 5 µm. (**c**), (**d**) AFM images of the ReS_2 flake in **(a)** and the corresponding $ReS_2[O_2]_x$ superlattice in **(b)**, respectively. Scale bars: 1 µm. PL (**e**) and Raman spectra (**f**) of the pristine ReS_2 flake and the corresponding $ReS_2[O_2]_x$ superlattice.

References

[1] Huang, Q. *et al.* First-principles study of O-BN: A sp^3-bonding boron nitride allotrope. *J. Appl. Phys.* **112**, 053518 (2012).

[2] Lee, B. & Rudd, R. E. First-principles calculation of mechanical properties of Si <001> nanowires and comparison to nanomechanical theory. *Phys. Rev. B* **75**, 195328 (2007).

[3] Kresse, G. & Joubert, D. From ultrasoft pseudopotentials to the projector augmented-wave method. *Phys. Rev. B* **59**, 1758-1775 (1999).

[4] Perdew, J. P., Burke, K. & Ernzerhof, M. Generalized gradient approximation made simple. *Phys. Rev. Lett.* **77**, 3865 (1996).

[5] Peng, B., Yu, G., Liu, X. et al. Ultrafast charge transfer in MoS_2/WSe_2 p–n heterojunction. *2D Mater.* **3**, 025020 (2016).

[6] Shim, J. *et al.* Thin-film transistors: high-performance 2D rhenium disulfide (ReS_2) transistors and photodetectors by oxygen plasma treatment. *Adv. Mater.* **28**, 6984-6984 (2016).

[7] Rahman, M., Davey, K. & Qiao, S. Z. Advent of 2D rhenium disulfide (ReS_2): fundamentals to applications. *Adv. Funct. Mater.* **27**, 1606129 (2017).

[8] Hafeez, M., Gan, L., Li, H. Q., Ma, Y. & Zhai, T. Y. Large-area bilayer ReS_2 film/multilayer ReS_2 flakes synthesized by chemical vapor deposition for high performance photodetectors. *Adv. Funct. Mater.* **26**, 4551-4560 (2016).

[9] Tongay, S. *et al.* Monolayer behaviour in bulk ReS_2 due to electronic and vibrational decoupling. *Nat. Commun.* **5**, 3252 (2014).

[10] Liu, E. *et al.* Integrated digital inverters based on two-dimensional anisotropic ReS_2 field-effect transistors. *Nat. Commun.* **6**, 6991 (2015).

[11] Feng, Y. *et al.* Raman vibrational spectra of bulk to monolayer ReS_2 with lower

symmetry. *Phys. Rev. B* **92**, 054110 (2015).

[12] Chenet, D. A. *et al.* In-plane anisotropy in mono- and few-Layer ReS_2 probed by raman spectroscopy and scanning transmission electron microscopy. *Nano Lett.* **15**, 5667-5672 (2015).
